# RIPK3 blockade attenuates tubulointerstitial fibrosis in a mouse model of diabetic nephropathy

**DOI:** 10.1038/s41598-020-67054-x

**Published:** 2020-06-26

**Authors:** Ying Shi, Chunling Huang, Yongli Zhao, Qinghua Cao, Hao Yi, Xinming Chen, Carol Pollock

**Affiliations:** 1 University of Sydney, Sydney Medical School, Kolling Institute of Medical Research Sydney, Sydney, NSW 2065 Australia; 2 The Second Affiliated Hospital of Dalian Medical University, Department of Pediatrics 467 Zhongshan Road, Shahekou District Dalian, Liaoning, CN 116027 China

**Keywords:** Chronic kidney disease, Renal fibrosis

## Abstract

Receptor-interacting protein kinase-3 (RIPK3) is a multifunctional regulator of cell death and inflammation. RIPK3 controls cellular signalling through the formation of the domain-like receptor family pyrin domain-containing 3 (NLRP3) inflammasome, which is recognised to mediate renal fibrogenesis. The role of RIPK3 in diabetic kidney disease (DKD) induced renal fibrosis has not been previously determined. To define the action of RIPK3 in the development of diabetic kidney disease, wild-type (WT), RIPK3 -/- and endothelium-derived nitric oxide synthase (eNOS)-/- mice were induced to develop diabetes mellitus using multiple low doses of streptozotocin and maintained for 24 weeks. RIPK3 activity and NLRP3 expression were upregulated and fibrotic responses were increased in the kidney cortex of WT mice with established diabetic nephropathy compared to control mice. Consistently, mRNA expression of inflammasome components, as well as transforming growth factor beta 1 (TGFβ1), α smooth muscle actin (α-SMA) and collagen deposition were increased in diabetic kidneys of WT mice compared to control mice. However, these markers were normalised or significantly reversed in kidneys of diabetic RIPK3 -/- mice. Renoprotection was also observed using the RIPK3 inhibitor dabrafenib in eNOS-/- diabetic mice as demonstrated by reduced collagen deposition and myofibroblast activation. These results suggest that RIPK3 is associated with the development of renal fibrosis in DKD due to the activation of the NLRP3 inflammasome. Inhibition of RIPK3 results in renoprotection. Thus, RIPK3 may be a potential target for therapeutic intervention in patients with diabetic kidney disease.

## Introduction

End-stage kidney disease (ESKD) is a major cause of morbidity and mortality in patients with diabetes mellitus. Renal fibrosis is characteristic of most, if not all, forms of chronic kidney disease (CKD). The increasing prevalence of diabetes mellitus accounts for the majority of chronic kidney disease worldwide^1^. The mainstay of therapy for diabetic kidney disease (DKD) is currently limited to controlling blood glucose and blood pressure, generally with an agent that blocks the renin-angiotensin system^2^ and more recently inhibition of the sodium-glucose linked co-transporter (SGLT)-2^3^. To date, no specific therapy for preventing diabetic kidney disease is available. A successful continuum between innovative discovery science and rigorous translation of research findings is required to improve the outcomes of patients with diabetic kidney disease.

Many types of kidney injury cause kidney inflammation derived from invading immune cells as well as intrinsic renal cells, with the consequent release of profibrotic cytokines that drive the fibrotic process. Limiting kidney inflammation is important in halting the progression of CKD. The receptor-interacting protein kinase (RIPK)3, a crucial kinase mediating necroptosis, has been increasingly implicated as a potential regulator of kidney inflammation^4–6^. Deletion either RIPK3 or the substrate of RIPK3 in the necroptosis pathway, mixed-lineage kinase domain-like (MLKL) resulted in reduced kidney damage in an oxalate crystal-induced acute kidney injury mouse model^7^ and kidney ischemia-reperfusion injury mouse model^8^. However, in folic acid-induced AKI and unilateral ureteral obstruction–induced renal fibrosis, blockade of MLKL, failed to protect against fibrogenesis or kidney injury while mice with RIPK3 deficiency showed reduced renal fibrosis and inflammatory response^5,9^, which indicates a necroptosis-independent role of RIPK3. Furthermore, RIPK3 was found to promote NLRP3 inflammasome and IL-1β inflammatory responses independent of MLKL and necroptotic cell death^10^. Our pilot study also shows that phosphorylated level of MLKL does not change in the diabetic mouse model (supplementary result). However, the function of RIPK3 in the development of fibrogenesis remains largely unknown.

The domain-like receptor family pyrin domain-containing (NLRP)3 inflammasome has been well established in various models of kidney disease, including DKD^11,12^. The NLRP3 inflammasome promotes renal tubular epithelial cell injury and interstitial fibrosis mainly through the biological function of inflammasome induced cell injury, transforming growth factor-beta (TGFβ) signalling, and tubular cell epithelial-mesenchymal transition (EMT)^13^. RIPK3 has been implicated as a regulator of NLRP3 inflammasome signalling in macrophages^10^. However, the function of RIPK3 mediated NLRP3 inflammasome signalling in renal tubular cells has not been elucidated.

In this study, we examined the role of RIPK3 in DKD induced renal fibrosis using a streptozotocin (STZ)-induced diabetic mouse model. We found that RIPK3 deficiency attenuated diabetes-induced renal fibrosis, in association with reduced activation of the NLRP3 inflammasome. Dabrafenib treatment also attenuated diabetes-induced collagen deposition and myofibroblast activation. Our data support the tenet that RIPK3 may mediate diabetes-induced fibrosis through the NLRP3 inflammasome. Hence strategies that include inhibition of RIPK3 may limit the development of diabetic kidney disease.

## Results

### Upregulation of RIPK3 and NLRP3 expression in the diabetic kidney

**Figure 1:**
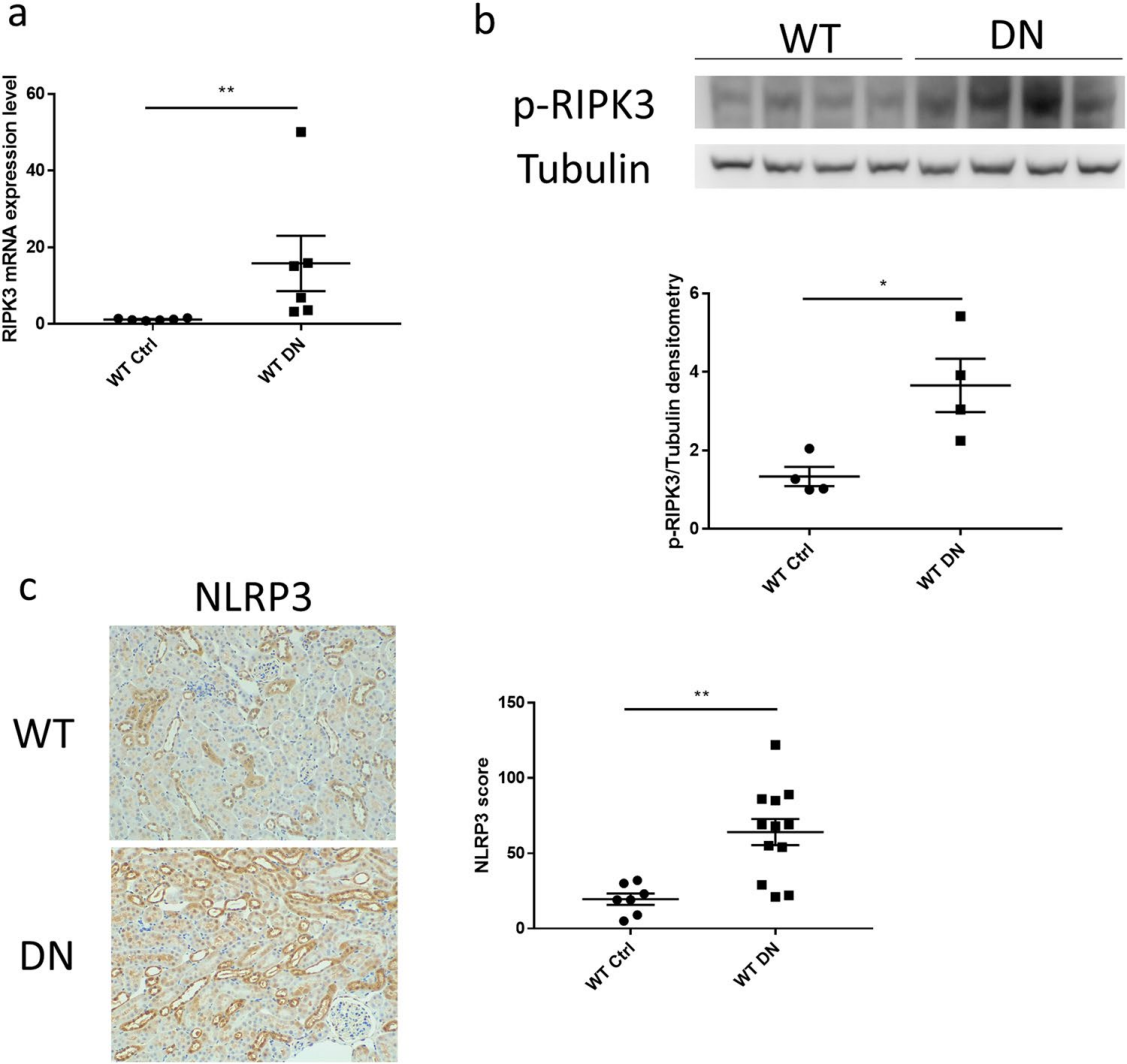
Increased expression of receptor-interacting protein kinase (RIPK) 3 and pyrin domain-containing protein (NLRP) 3 in diabetic kidney disease. (**a**) was reused from the thesis^14^. (**a**) RIPK3 mRNA expression fold change was measured by RT-PCR in mice (wild-type) +/− diabetes mellitus. Tubulin was used as the reference gene. (**b**) Immunoblot analysed of kidneys from the same groups as in (**a**) using antibodies for phospho-RIPK3 and Tubulin. (**c**) Representative immunohistochemical staining images and the quantitation of NLRP3 expression in the renal cortex from the same groups as in (**a**). Magnification: 20×. Statistical analysis was performed using two-tailed *t*-tests. For all graphs, each dot or triangle represents an individual sample, and horizontal bars denote the mean ± S.E.M. *P < 0.05, **P < 0.01.

To determine whether RIPK3 is upregulated in the diabetic kidney, mRNA expression of RIPK3 was assessed using RT-PCR. As shown in Fig. 1a (Fig. 1a was reused from the thesis^14^), RIPK3 mRNA expression was significantly increased in WT diabetic mice compared to non-diabetic controls (P < 0.01). Phosphorylation of RIPK3 (p-RIPK3) was examined using western blot in kidneys of WT mice with or without diabetic nephropathy. Consistent upregulation of p-RIPK3 protein was observed in the kidneys of diabetic WT mice compared to control kidneys (P < 0.05, Fig. 1b). Our data indicate that RIPK3 activity is upregulated within the kidney in the context of diabetic kidney disease.

To identify the expression of the NLRP3 inflammasome, NLRP3 protein expression in kidney cortex was examined using immunohistochemistry. As shown in Fig. 1c, upregulation of NLRP3 was observed in tubular cells within the kidneys of diabetic WT mice compared to control kidneys (P < 0.01). We also assessed the NLRP3 expression in glomeruli from the control group and the diabetic group. No significant change was found by comparing control mice with diabetic mice (supplementary result). Therefore, NLRP3 induced kidney injury in DKD is likely to be mainly in the tubulointerstitial compartment.

### RIPK3 gene knockout reduced NLRP3 inflammasome formation

**Figure 2:**
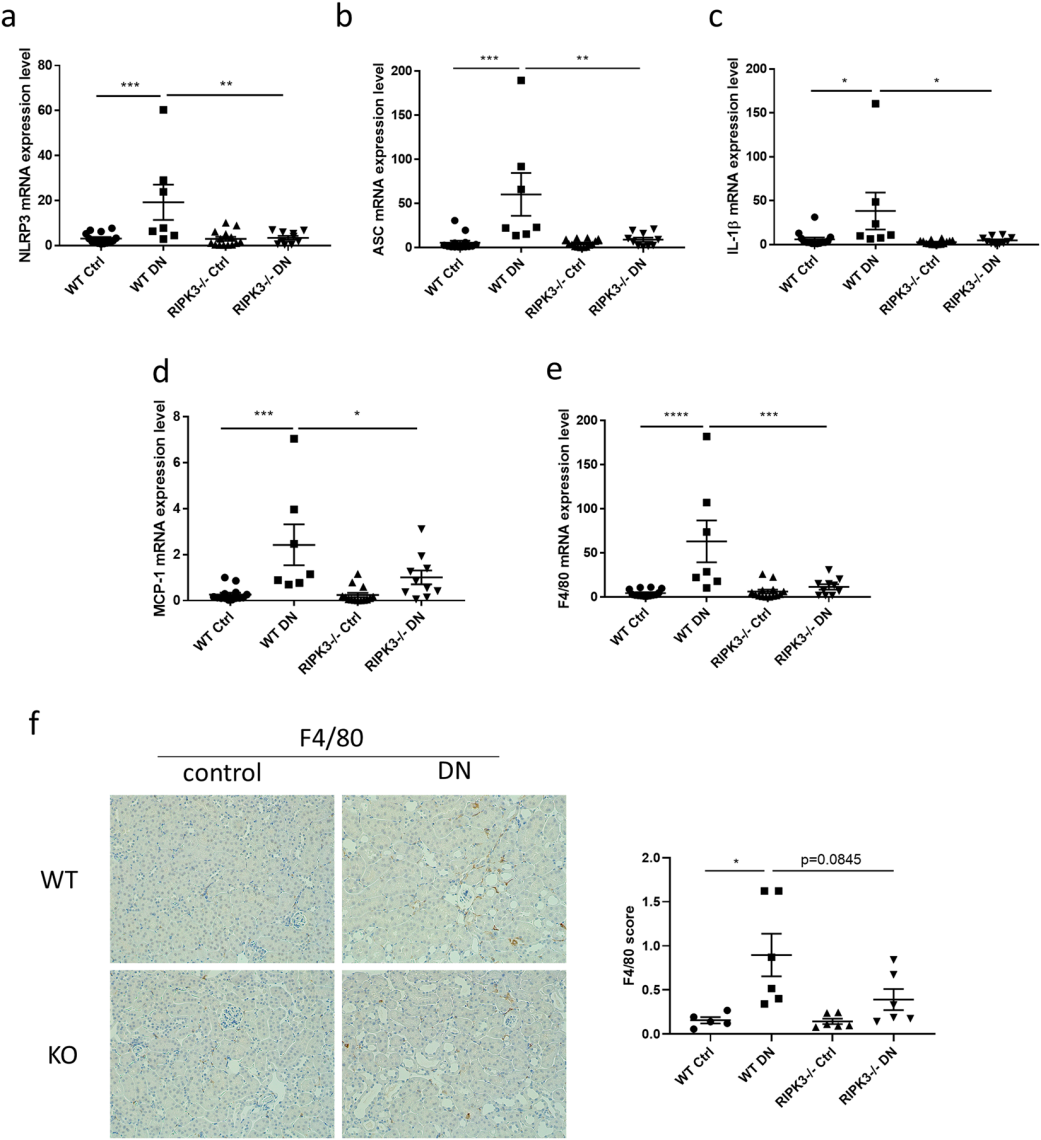
Blockade of RIPK3 alleviated the formation of NLRP3 inflammasome and inflammatory response in kidneys of diabetic mice. **(a**–**e**) were reused from the thesis^14^. (**a**–**e**) NLRP3, adapter protein apoptosis-associated speck-like protein (ASC), interleukin (IL)-1β, monocyte chemoattractant protein (MCP)-1 and F4/80 mRNA expression were measured by RT-PCR in mice (WT or RIPK3−/−) + /− diabetic nephropathy. Tubulin was used as the reference gene. (**f**) Representative immunohistochemical staining images and the quantitation of F4/80 expression in the renal cortex from the same groups as in (**a**). Magnification: 20×. Statistical analysis was performed by one-way analysis of variance ANOVA followed by Tukey’s multiple comparisons test. For all graphs, each dot or triangle represents an individual sample, and horizontal bars denote the mean ± S.E.M. *P < 0.05, **P < 0.01. ***P < 0.001. ****P < 0.0001.

To determine if RIPK3 regulates NLRP3 activation in the kidneys of diabetic mice, mRNA expression of the NLRP3 inflammasome components NLRP3, the adapter protein apoptosis‐related speck‐like protein (ASC) and the downstream cytokine interleukin (IL)-1β were examined using RT-PCR. As shown in Fig. 2a (Fig. 2a reused from the thesis^14^), mRNA expression of NLRP3 was upregulated in kidneys of diabetic WT mice compared to non-diabetic controls (P < 0.001), and as hypothesised, this was completely reversed in RIPK3 gene knockout mice (Fig. 2a, P < 0.01). ASC mRNA expression was significantly elevated in the kidney of diabetic WT mice compared to non-diabetic controls, whereas RIPK3 gene knockout animals also showed normalisation of ASC mRNA expression in diabetic kidneys (all P < 0.05, Fig. 2b, Fig. 2b was reused from the thesis^14^). IL-1β mRNA was elevated in kidneys of diabetic WT mice and gene ablation of RIPK3 reversed this effect (all P < 0.05, Fig. 2c, Fig. 2c was reused from the thesis^14^). Together, these data indicate that loss of RIPK3 reduces NLRP3 formation and activation within the diabetic kidney cortex.

### RIPK3 gene knockout reduced kidney inflammatory cell infiltration

It is well known that inflammatory cell infiltration plays a crucial role in progressive diabetic kidney disease. Thus, we measured monocyte chemoattractant protein (MCP)-1 and the mRNA expression of the macrophage marker F4/80. As shown in Fig. 2d (Fig. 2d was reused from the thesis^14^), MCP-1 mRNA expression was up-regulated in kidneys of diabetic WT mice compared to non-diabetic controls. This upregulation was partially reversed in diabetic RIPK3 -/- mice (all P < 0.05). Similarly, F4/80 mRNA expression was increased significantly in the kidney cortex of diabetic WT mice compared to non-diabetic controls, whereas F4/80 mRNA expression in kidneys of diabetic RIPK3-/- mice was much lower compared to diabetic WT mice (all P < 0.05, Fig. 2e, Fig. 2e was reused from the thesis^14^). Consistently, the F4/80 stained macrophages were increased in the renal interstitium of diabetic WT mice compared to non-diabetic controls (P < 0.05, Fig. 2f), while RIPK3 blockade showed a trend to reduce the macrophage infiltration (P = 0.0845, Fig. 2f). Collectively, these data indicate that loss of RIPK3 contributed to the suppression of inflammatory cell infiltration in the diabetic kidney cortex.

### RIPK3 gene knockout decreased TGFβ1 expression and myofibroblast activation

**Figure 3:**
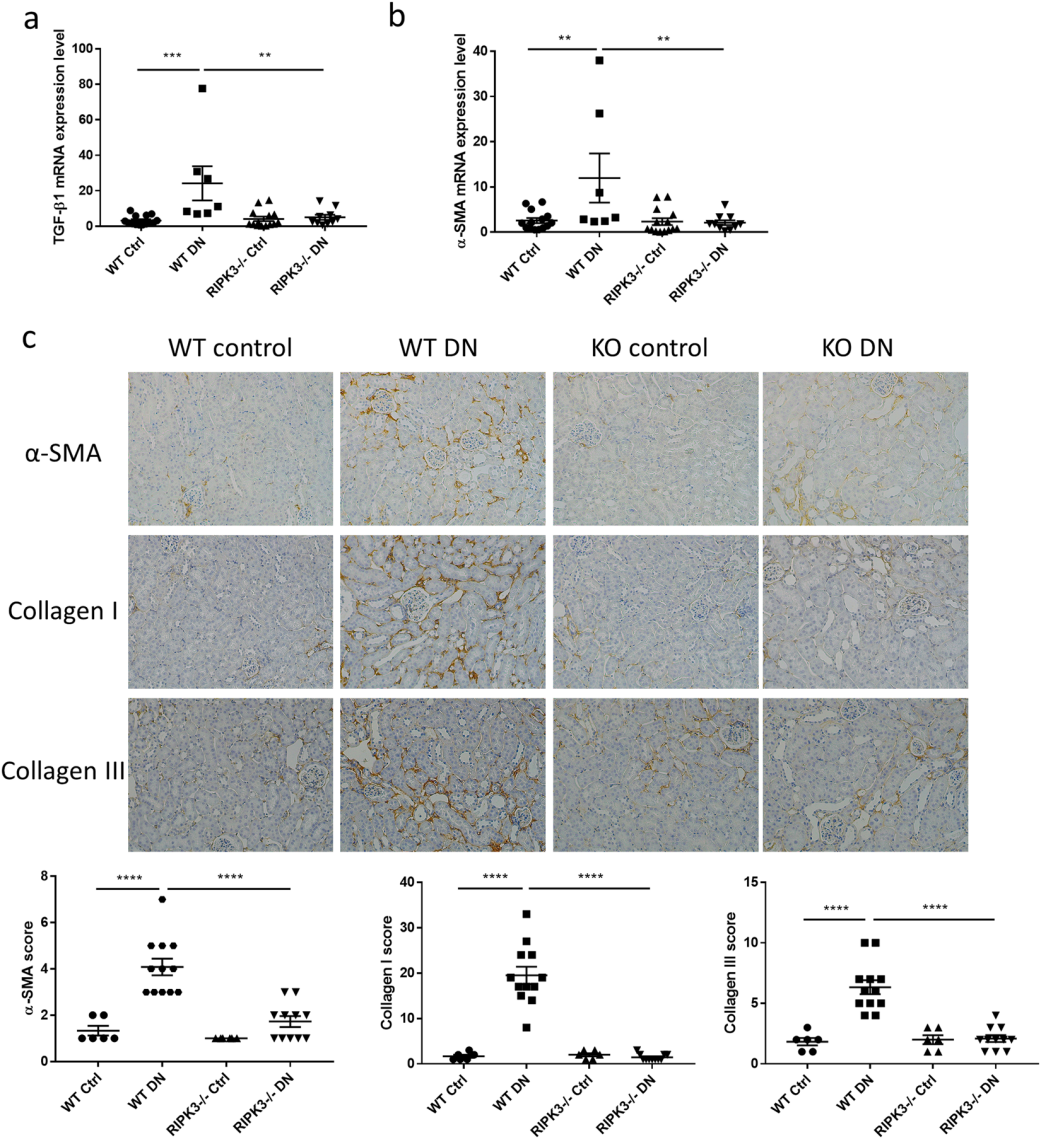
Blockade of RIPK3 alleviated fibrotic response in kidneys of diabetic mice. (**a,b**) were reused from the thesis^14^. (**a,b**) transforming growth factor beta (TGFβ) 1 and α-smooth muscle actin (α-SMA) were measured in mice (wild-type WT or RIPK3−/−) + /− diabetes mellitus by RT-PCR. (**c**) Representative immunohistochemical staining images and the quantitation of α-SMA, collagen I and collagen III expression in the renal cortex from the same groups as in (**a**). Magnification: 20×. Statistical analysis was performed with ANOVA followed by Tukey’s multiple comparisons test. For all graphs, each dot or triangle represents an individual sample, and horizontal bars denote the mean ± S.E.M. **P < 0.01. ***P < 0.001. ****P < 0.0001.

To understand the association between RIPK3 and myofibroblast activation we examined renal TGFβ1 mRNA expression levels in diabetic WT and RIPK3 -/- mice using RT-PCR. Induction of diabetes in WT mice led to significantly increased TGFβ1 mRNA expression (P < 0.001, Fig. 3a, Fig. 3a was reused from the thesis^14^), while TGFβ1 gene expression was reduced in RIPK3 -/- mice (P < 0.01, Fig. 3a). α-smooth muscle actin (α-SMA) gene expression is commonly regarded as a specific marker of myofibroblast differentiation downstream of TGFβ1 activation. Hence α-SMA mRNA was examined using RT-PCR. The data showed that α-SMA mRNA expression levels in kidneys of WT mice with diabetes were upregulated compared to non-diabetic mice (P < 0.01, Fig. 3b, Fig. 3b was reused from the thesis^14^). Its expression levels were normalized in kidneys of diabetic RIPK3 -/- mice (P < 0.01, Fig. 3b). The expression of α-SMA was also examined using immunohistochemistry to determine the myofibroblast activation in renal interstitium. α-SMA was consistently upregulated in the renal cortex of diabetic WT mice compared to control kidneys and reduced by RIPK3 deficiency (P < 0.0001, Fig. 3c). These data suggest that disruption of RIPK3 suppresses TGFβ1 expression and myofibroblast differentiation in kidneys.

### RIPK3 gene knockout reduced collagen I and III deposition induced by diabetes

To investigate whether RIPK3 deficiency alleviates renal fibrogenesis, we compared WT and RIPK3 -/- mice, each with or without diabetes. The expression of the collagen I and III were measured in the kidney cortex of mice by immunohistochemical analysis. As shown in Fig. 3c elevated collagen I was found in diabetic WT mice compared to non-diabetic controls (P < 0.0001). However, collagen I expression levels in diabetic RIPK3-/- mice were significantly lower than that in the diabetic WT mice (P < 0.0001, Fig. 3c). Similarly, type III collagen expression was up-regulated in kidneys from diabetic WT mice compared to non-diabetic WT control (P < 0.0001, Fig. 3c). RIPK3 gene knockout reversed diabetes-induced type III collagen expression in the kidney cortex compared to diabetic WT mice (P < 0.0001, Fig. 3c).

### **Dabrafenib treatment increases survival rate in mice with established diabetic nephropathy**

**Figure 4:**
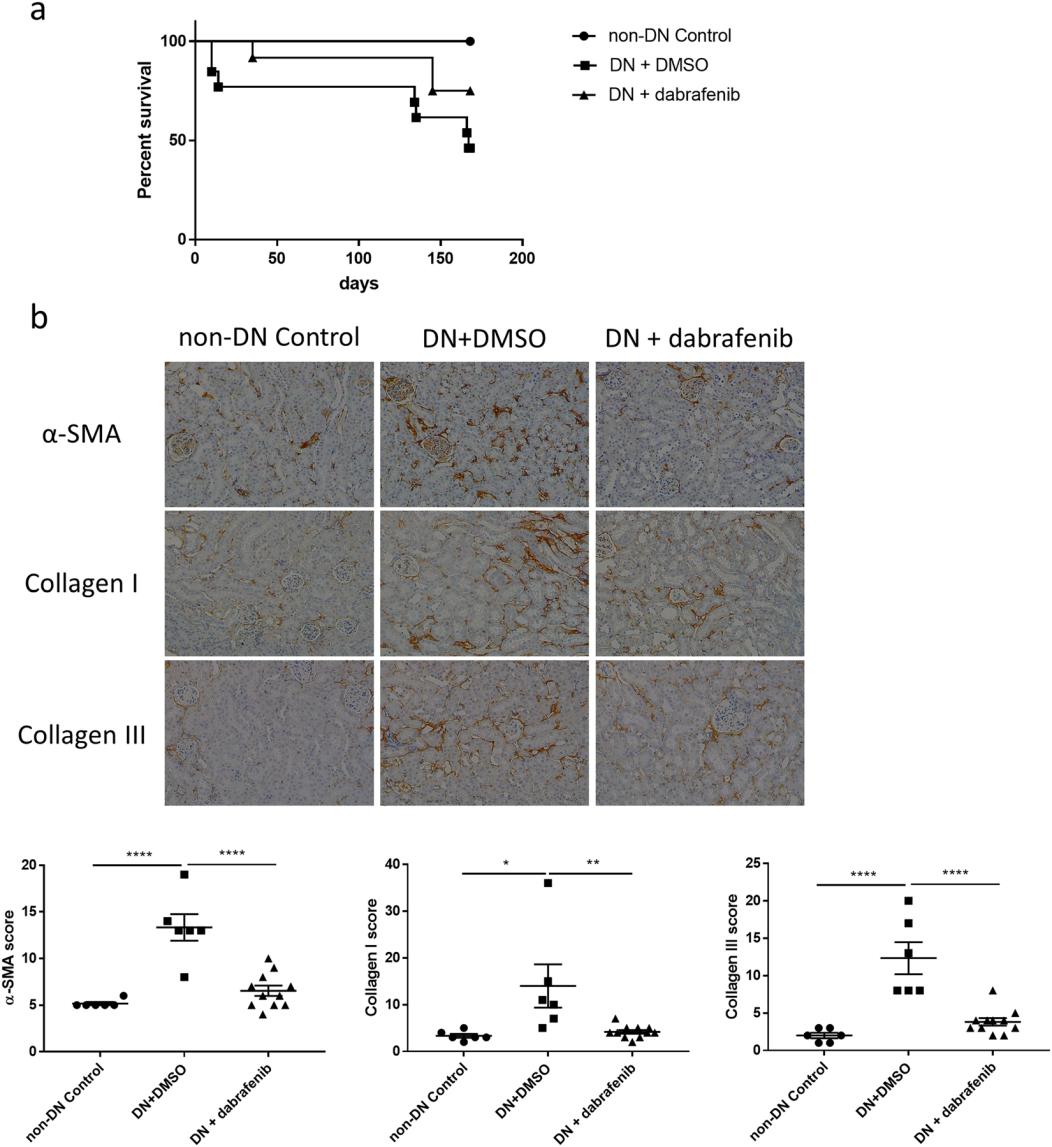
Dabrafenib reduced the fibrotic response in the kidneys of diabetic mice. (**a**,**b**) were reused from the thesis^14^. (**a**) Survival curves of eNOS−/− mice (with or without treatment) +/− diabetic nephropathy. The survival curves were analyzed by the Log-rank (Mantel-Cox) test. (**b**) Representative immunohistochemical staining images and the quantitation of α-SMA, collagen I and collagen III expression in the renal cortex from the same groups as in (**a**). Magnification: 20×. Statistical analysis was performed with ANOVA followed by Tukey’s multiple comparisons test. For all graphs, each dot or triangle represents an individual sample, and horizontal bars denote the mean ± S.E.M. *P < 0.05, **P < 0.01. ****P < 0.0001.

To study whether the RIPK3 inhibitor dabrafenib suppresses the progression of diabetic nephropathy and associated mortality, survival rates of eNOS-/- mice after induction of diabetes were assessed over 24 weeks. As shown in Fig. 4a (Fig. 4a was reused from the thesis^14^), all control mice survived throughout the experimental period. Diabetic mice, without dabrafenib treatment, had a significantly decreased survival compared with the control group (P = 0.0015), reflecting a survival rate of 84.6% at day 10, decreasing to 76.9% at day 14. Further attrition occurred at the 134^th^, 135^th^ day, 166^th^ day and 167^th^ day) resulting in a 46.2% survival at the time of culling. However, dabrafenib treatment conferred an increased survival rate at each time point compared to untreated mice reflected in a survival rate of 75% after 24 weeks of diabetes. This data demonstrates that dabrafenib confers a survival benefit in animals with diabetic kidney disease.

### **Dabrafenib treatment reduces myofibroblast activation and collagen deposition in mice with established diabetic nephropathy**

To determine if dabrafenib alleviates diabetes-induced renal fibrosis, we measured the α-SMA protein expression within the kidney cortex to investigate if dabrafenib reduces myofibroblast activation. As shown in Fig. 4b (Fig. 4b was reused from the thesis^14^), diabetic mice showed significantly elevated α-SMA protein expression, whereas this effect was significantly reduced in mice exposed to dabrafenib (P < 0.0001). We also performed immunohistochemistry to measure collagen deposition in the kidney. As shown in Fig. 4b, diabetic mice had significantly higher renal cortical collagen I expression (P < 0.05), whereas this increase was reversed by dabrafenib treatment (P < 0.01). Similarly, diabetes-induced an up-regulation of renal cortical collagen III (P < 0.0001), which was largely abrogated in dabrafenib treated mice (P < 0.0001, Fig. 4b). Thus, diabetes-induced myofibroblast activation and collagen accumulation in the kidney was attenuated by dabrafenib treatment.

### Diabetes-induced albuminuria is unaffected by blockade of RIPK3

**Figure 5:**
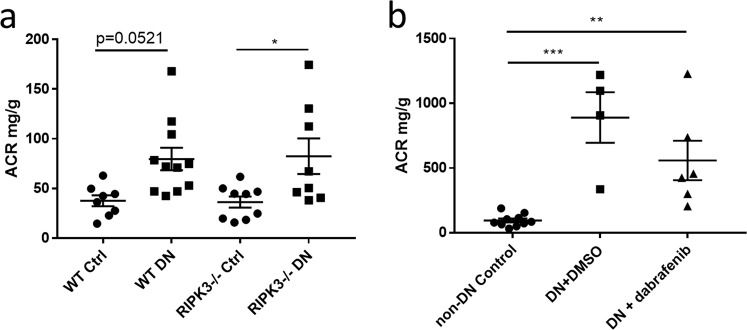
Blockade of RIPK3 did not reduce the diabetes-induced albuminuria. (**a**) Albumin creatinine ratio (ACR) was measured in mice (wild-type WT or RIPK3−/−) + /− diabetes mellitus. (**b**) ACR of eNOS−/− mice (with or without treatment) +/− diabetic nephropathy was measured by ELISA. Statistical analysis was performed with ANOVA followed by Tukey’s multiple comparisons test. For all graphs, each dot or triangle represents an individual sample, and horizontal bars denote the mean ± S.E.M. *P < 0.05, **P < 0.01. ***P < 0.001.

Having demonstrated that both RIPK3 gene knockout and dabrafenib improved the fibrotic response induced by diabetes, we assessed the albuminuria with albumin-to-creatinine ratio (ACR). As shown in Fig. 5a, diabetes caused a two-fold increase in urinary albumin excretion while a similar increase was also observed in the diabetic mice treated with the vehicle of dabrafenib (Fig. 5b). However, neither RIPK3-/- or dabrafenib treated mice showed a significant reduction in diabetes-induced albuminuria (Fig. 5a,b). We also assessed the glomerulosclerosis in glomeruli from the control group and the diabetic group. Diabetes induced significant glomerulosclerosis in the kidney. However, RIPK3 gene knockout failed to reduce the glomerulosclerosis (supplementary result), that might explain the lack of effect in ACR by RIPK3 blockade.

## Discussion

This study was undertaken to define the role of RIPK3 in regulating tubulointerstitial fibrosis in diabetic nephropathy. Significant up-regulation of RIPK3 activity was evident in the kidney cortex of animals with diabetes. RIPK3 deficiency downregulated NLRP3 inflammasome activation, associated with a reduced inflammatory cell infiltration, myofibroblast activation and less collagen deposition. The RIPK3 inhibitor dabrafenib replicated these renoprotective effects.

The NLRP3 inflammasome, a crucial source of IL-1β, contributes to the progression of renal fibrosis^13^. However, tubular secretion of IL-1 is still under debate^15–17^. We measured IL-1β expression in the kidney cortex (supplementary result) by western blot. No significant change was found between WT control and WT diabetic animals. This result supports that IL-1 β in the active form may not reflect tubular injury via the NLRP3 inflammasome.

It has been reported that NLRP3 deletion protects against renal fibrosis and attenuates inflammation in diabetic mice^18^ and mice with 5/6 nephrectomy^19^. In the present study, we found genetic deletion of RIPK3 reduced NLRP3 inflammasome activation, associated with reduced fibrotic response and inflammatory cell filtration. Thus, we conclude the benefit of RIPK3 inhibition on inflammatory response may arise from inhibition of NLRP3 activation. It is well documented that activation of NLRP3 inflammasome triggers EMT induced by high glucose or TGFβ1^20^, which serves to amplify the fibrotic response^21,22^. In our study, both RIPK3 knockout and inhibition abolished the myofibroblast activation and the associated collagen deposition. Hence it is likely that RIPK3 may promote EMT as a consequence of activation of the NLRP3 inflammasome.

It is well recognised that TGFβ1 regulates the activation of myofibroblasts and renal fibrotic responses. Our data elucidate that deletion of RIPK3 reduces TGFβ1 mRNA expression, myofibroblast activation and collagen accumulation. It has been recently being independently reported that RIPK3 is implicated in renal fibrogenesis in two alternative models of renal fibrosis ie the unilateral ureteral obstruction (UUO) and adenine-induced models of CKD^5^. Similarly, they found that inhibition of RIPK3 suppressed extracellular matrix production and myofibroblast differentiation. However, RIPK3 did not alter the TGFβ1 mRNA expression at 7 days in the UUO mice^5^ when renal fibrogenesis is likely to have been instigated in the model. Hence it is likely that RIPK3 can upregulate multiple pathways involved in renal fibrogenesis, which may differ between cell types and depend on the strength of the fibrogenic stimuli.

In our study, RIPK3 inhibition prevented fibrosis in diabetic nephropathy but failed to improve diabetes-induced albuminuria. It is well accepted that albuminuria is the result of damage to an essentially impermeable glomerular barrier and further dysfunction in the proximal tubular cells^23^. Our data support that ablation of RIPK3 alleviates tubulointerstitial fibrosis by downregulating TGF-β1 mRNA expression, myofibroblast activation and collagen accumulation in the context of injured tubular cells, which is initiated by diabetes mellitus^24^. Collectively, RIPK3 may exert differential effects in the tubulointerstitial vs glomerular compartments.

In summary, the results from these animal studies have demonstrated that RIPK3 is an important mediator of tubulointerstitial fibrosis in diabetic kidney disease. Furthermore, we have shown that blockade of RIPK3 exerts antifibrotic effects in the kidney that are likely to be due to the inhibition of NLRP3 inflammasome activation.

## Methods

### Animal studies

6-8-week-old male WT, RIPK3-/-, endothelium-derived nitric oxide synthase (eNOS)-/- mice (all C57BL/6 background) weighing 20–25 g were used for this study. Mice were assigned to receive either 55 mg/kg STZ (Sigma-Aldrich) diluted in 0.1 M citrate buffer, pH 4.5, or citrate buffer alone by intraperitoneal injection for five consecutive days as described previously^25^. STZ-treated animals with blood glucose>16 mmol/L were considered as diabetic. Dabrafenib was used as the RIPK3 inhibitor in our study. Previous studies have reported that dabrafenib is a RIPK3 inhibitor in various models, including human hepatocytes^26^, mouse models of acetaminophen-induced liver injury^26^ and ischemic brain injury^27^. In addition, dabrafenib is a well-known inhibitor of B-Raf, which suppresses the downstream Ras/Raf/ERK/MAPK pathway^28^ which has been approved for clinical use for the treatment of non-small cell lung cancer expressing B-Raf^V600E^ mutations and in melanoma^29^. Inhibition of Raf kinase is known to attenuate renal fibrosis in a rat model of autosomal dominant polycystic kidney disease^30^. It is also recognised that TGFβ 1 can induce Ras/Raf/ERK/MAPK pathways^31^, resulting in renal fibrosis. Therefore, compared to the most widely-used RIPK3 inhibitor GSK’872^32^, dabrafenib may show renoprotection in the context of diverse models of renal fibrosis. eNOS-/- mice were used to study the effect of dabrafenib on the development of diabetic nephropathy. Alzet model 2006 osmotic pumps (0.15 µl/hr of dabrafenib dissolved in vehicle dimethyl sulfoxide (DMSO) at a concentration of 16.7 mg/ml) were implanted in the subcutaneous tissues in the interscapular region of eNOS-/- diabetic mice to deliver dabrafenib or DMSO. Mice were weighed, and the blood glucose was monitored weekly with an ACCU-CHEK glucometer (Roche Diagnostics). Insulin (Lantus 2U/ day) was given if the blood glucose of mice exceeded 28 mmol or mice had significant weight loss (10–15% body weight loss in mouse with blood glucose>16 mmol/L). A further 0.5 units was given for every further increase of 10 mmol glucose beyond 28 mmol/L. The quantification of insulin doses throughout the study has been included in supplementary file. Mice were sacrificed 24 weeks after the induction of diabetes. A pre-terminal 24-h urine was collected, and then the urine albumin and creatinine levels were measured with ELISA kits.

All animals were housed in the Kearns Animal Facility of the Kolling Institute of Medical Research, with a stable environment maintained at 22 ± 1 °C with a 2/12 h light-dark cycle. Experiments were conducted following the National Health and Medical Research Council of Australia’s Code for the Care and Use of Animals for Scientific Purposes and were approved by the Animal Research Ethics Committee of the Royal North Shore Hospital (NSLHD reference RESP/15/206).

### Western blot analysis

Mouse kidney cortex tissues were homogenized and lysed in RIPA buffer with protease and phosphatase inhibitors (ThermoFisher). Tissue lysates were separated on 4–12% gels (Invitrogen) and then transferred to PVDF membrane (PALL) for 2 hours at 4 °C. Membranes were blocked with 5% BSA in TBS-T (20 mM Tris, 150 mM NaCl, 0.1% Tween 20, pH 7.6) for 1 hour and then incubated with primary antibodies (Tubulin, T906, Sigma; phospho-RIPK3, ab56164, Abcam; MLKL, #37705, cell signalling technology; phospho-MLKL, #62233, Cell Signalling; IL-1, Ab9722, Abcam) at 4 °C overnight followed by relevant horseradish peroxidase-conjugated secondary antibody (anti-Rabbit antibody, Cell Signalling; anti-Mouse antibody, Santa Cruz). The membranes were then detected using ECL (Millipore) with LAS-4000 Imaging System (Fujifilm). Gel-pro software was used to analyse the densitometry of immunoblot images.

### RNA isolation and RT-PCR analysis

Total RNA was extracted from mouse kidney cortex using the RNeasy plus mini kit (Qiagen) by QIAcube (Qiagen). cDNA was synthesized with a cDNA synthesis kit (Roche). Predesigned probes (PrimeTime® Assay Std Probe 5’ 6-FAM™/ZEN™/3’ IB®FQ, Integrated DNA Technologies), including RIPK3 (Assay ID: Mm.PT.58.12712973.gs), TGFβ1 (Assay ID: Mm.PT.58.11254750), α-SMA (Assay ID: Mm.PT.58.16320644), MCP-1 (Assay ID: Mm.PT.58.42151692), F4/80 (Assay ID: Mm.PT.58.11087779), NLRP3 (Assay ID: Mm.PT.58.13974318), ASC (Assay ID: Mm.PT.56a.42872867), IL-1 β (Assay ID: Mm.PT.58.41616450), Tubulin (Assay ID: Mm.PT.58.13069923) were measured. Tubulin was used as the endogenous reference gene.

### Histology and Immunohistochemistry

Kidneys were fixed in 4% paraformaldehyde and embedded with paraffin. 4 μm kidney sections were cut and stained as follows. After deparaffinisation with xylene, slides were immersed in decreasing concentration of ethanol (100%, 100%, 95%, 70%) and rinsed in a container of running water. 1% periodic acid solution (PER50%/100, POCD Scientific) and Schiff’s reagent (SCH500, POCD Scientific) was used for Periodic Acid-Schiff (PAS) staining.

Kidney tissues were fixed in 4% paraformaldehyde and embedded with paraffin. 4 μm kidney sections were cut and stained as follows: After deparaffinisation with xylene, slides were immersed in decreasing concentration of ethanol (100%, 100%, 95%, 70%) and rinsed in a container of running water. Citrate buffer at pH 6 was heated to 99 °C for epitope retrieval. Slides were placed in hot retrieval buffer for 20 minutes incubation followed by 20 minutes cooling to room temperature. Slides were blocked for 15 minutes (protein block serum-free ready to use x0909, Dako). Slides were incubated overnight at 4°C with primary antibodies as follows: NLRP3 (Abcam, ab214185, 1:250), type I collagen (Abcam, ab34710, 1:750), type III collagen (Abcam, ab7778, 1:500), a-SMA (Sigma-Aldrich, A2547, 1:10000), F4/80 (Abcam, ab111101, 1:100). Slides were then washed with TBS-T (50 mM Tris, 150 mM NaCl, 0.05% Tween 20, pH 7.6), and incubated with associated secondary antibodies (EnVision + system-HRP labelled polymer anti-rabbit k4003, Dako) for 30 minutes at room temperature. After washing with TBS-T, kidney sections were covered with DAB (liquid DAB + substrate chromogen system k3468, Dako) for 10 minutes. All experiments included controls without primary antibody. Immunohistochemical staining was scored by multiplying the percentage value of positive signals by the intensity. The score of intensity was from 1 to 5. Positive signals in the renal cortex regions and glomeruli were identified and quantified using Image J software.

### Statistical analysis

The results are presented as mean ± S.E.M. The differences between two groups were analysed by two-tailed *t*-tests, and comparison of multiple groups was analysed by one-way analysis of variance ANOVA followed by Tukey’s multiple comparisons test. The survival curves were analysed by Log-rank (Mantel-Cox) test. A two-sided p-value <0.05 was considered statistically significant.

## Supplementary information

Supplementary Information.

## Data Availability

All data generated or analysed during this study are included in this published article (and its Supplementary Information files).
