# Supplementary material for: RIPK3 blockade attenuates tubulointerstitial fibrosis in a mouse model of diabetic nephropathy
Source: Sci Rep. 2020 Jun 26;10:10458. doi: 10.1038/s41598-020-67054-x (PMC7319952; doi:10.1038/s41598-020-67054-x)
Supplement: Supplementary file 1 — Supplementary Information. [file 41598_2020_67054_MOESM1_ESM.pdf]

RIPK3 blockade attenuates tubulointerstitial fibrosis in a mouse model of diabetic nephropathy

Ying Shi[1], Chunling Huang[1], Yongli Zhao[2], Qinghua Cao[1], Hao Yi[1], Carol Pollock[1*], Xinming Chen[1]

[1] University of Sydney, Sydney Medical School, Kolling Institute of Medical Research

Sydney, NSW 2065, Australia

[2] The Second Affiliated Hospital of Dalian Medical University, Department of Pediatrics

467 Zhongshan Road, Shahekou District Dalian, Liaoning, CN 116027

*Corresponding author, Carol A Pollock;

carol.pollock@sydney.edu.au

Supplementary Information

1. Uncropped blots

Uncropped P-RIPK3 blot

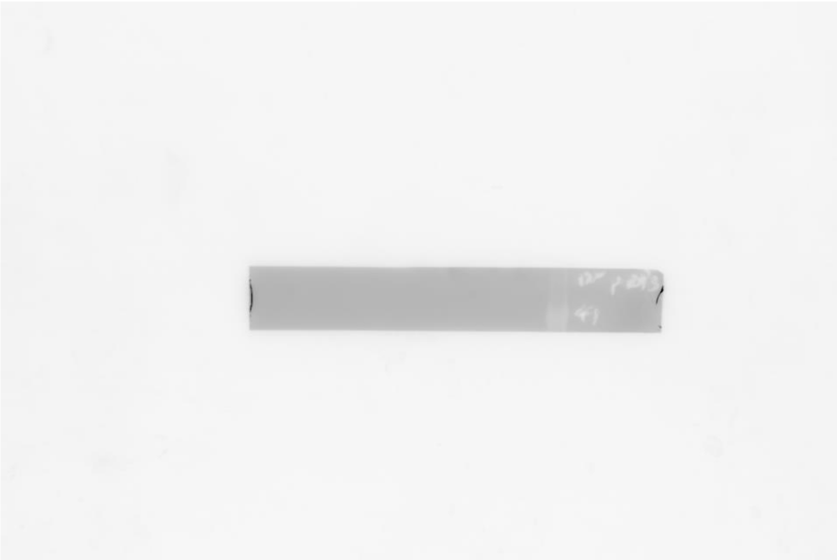

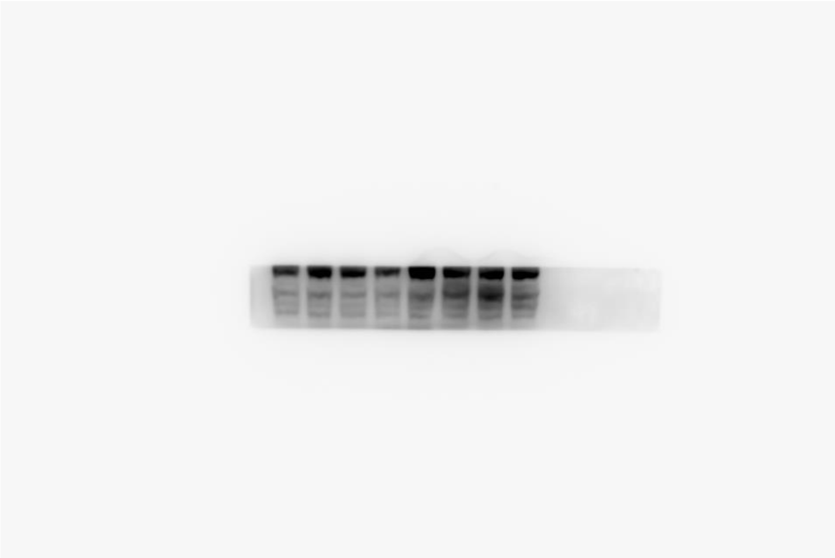

Uncropped tubulin blot

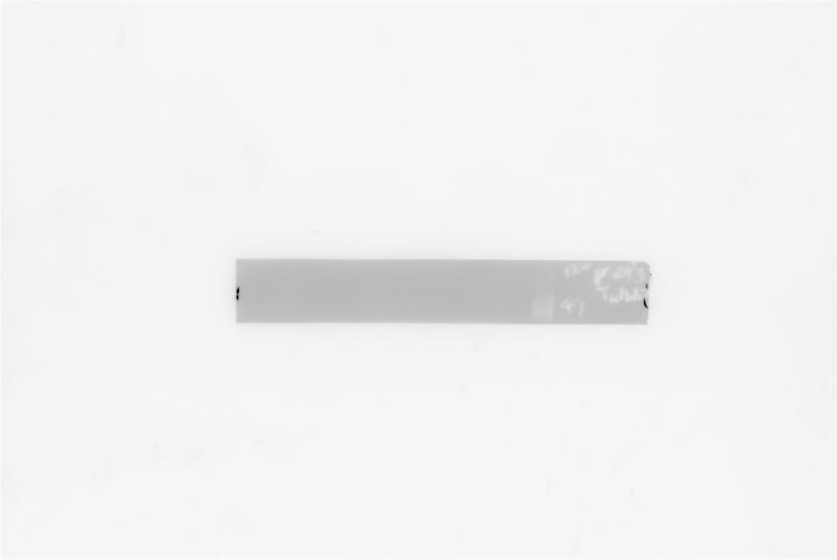

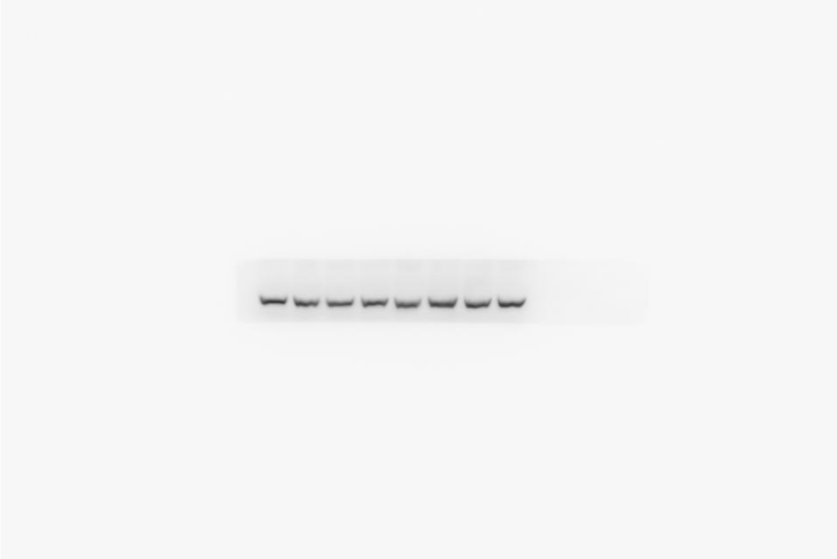

2. Supplementary data

Insulin doses administered to groups throughout the study

Groups	No. of mice/group	No. of mice needing insulin treatment	insulin doses throughout the study
WT Ctrl	14	-	-
WT DN	12	7	336 Units/mouse
RIPK3-/- Ctrl	14	-	-
RIPK3-/- DN	11	1	336 Units/mouse

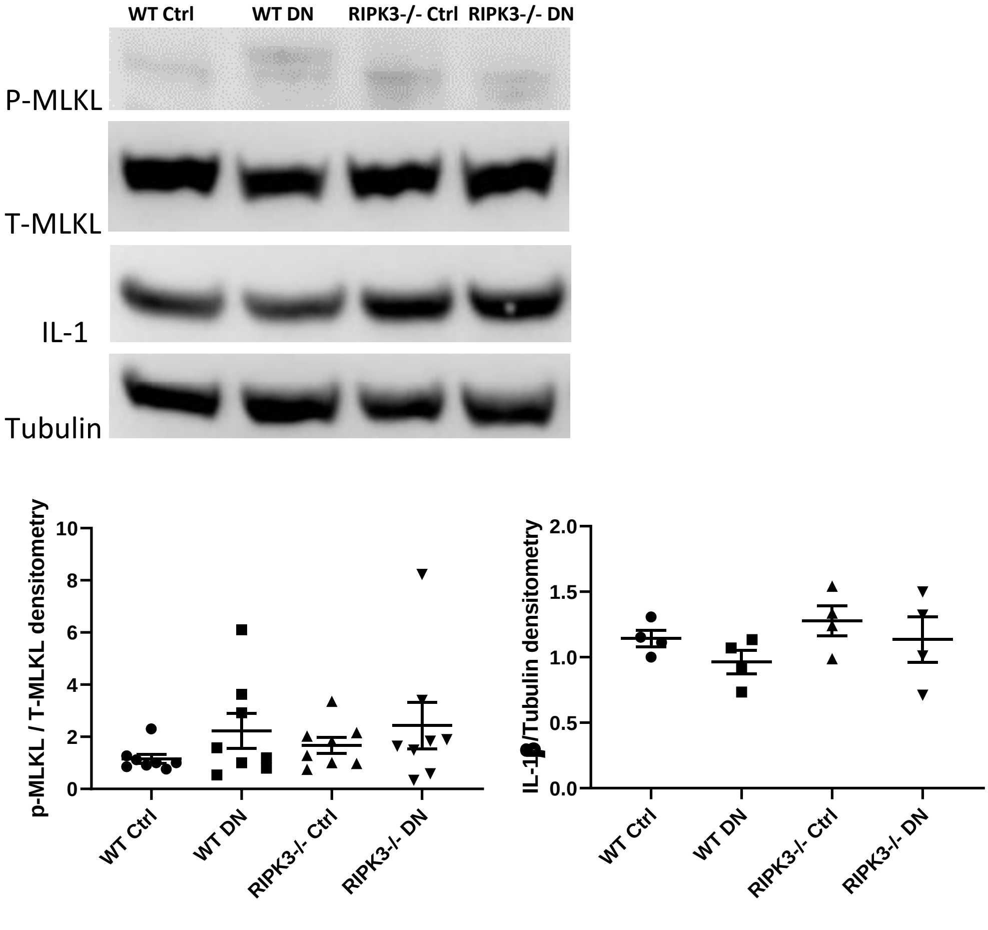

MLKL phosphorylation and IL-1β expression. Immunoblot analysed in kidneys from mice (wild-type WT or RIPK3-/-) +/- diabetes mellitus using antibodies for phospho-MLKL, Total MLKL, IL-1β and Tubulin. Statistical analysis was performed by one-way analysis of variance ANOVA followed by Tukey's multiple comparisons test. Each dot or triangle represents an individual sample, and horizontal bars denote the mean ± S.E.M.

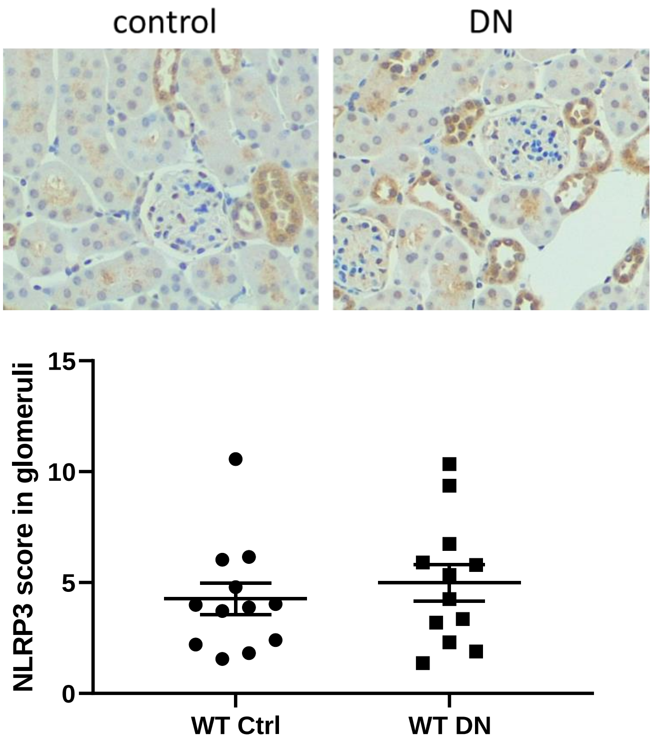

NLRP3 score in glomeruli. Immunohistochemical analysed in kidneys from mice (wild-type) +/- diabetes mellitus using antibody for NLRP3. Glomeruli were identified and circled by Image J software. 10-14 glomeruli have been selected for each kidney section. The positive signals in the selected glomeruli were quantified using Image J software. Immunohistochemical staining was scored by multiplying the percentage of positive signals by the intensity. Score of intensity was from 1 to 5. Magnification: 40×. Statistical analysis was performed using two-tailed *t*-tests. Each dot or triangle represents an individual sample, and horizontal bars denote the mean ± S.E.M.

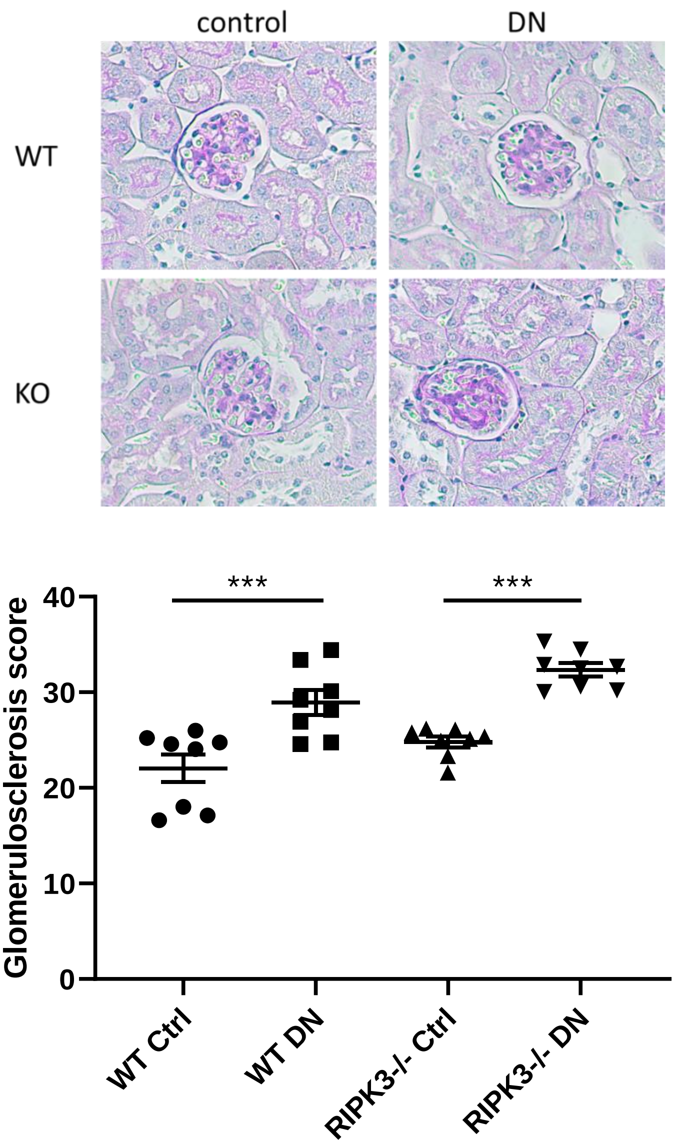

Glomerulosclerosis score. Periodic acid–Schiff (PAS)-stained kidney sections from mice (wild-type WT or RIPK3-/-) +/- diabetes mellitus were assessed. Glomeruli were identified and circled by Image J software. 10-14 glomeruli were selected for each kidney section. The positive signals in the selected glomeruli were quantified using Image J software and expressed as the score value. Magnification: 40×. Statistical analysis was performed using one-way ANOVA followed by Tukey's multiple comparisons test. Each dot or triangle represents an individual sample, and horizontal bars denote the mean ± S.E.M. ***, $P < 0.001$.
